# Emergence of Carbapenem-Resistant *Klebsiella pneumoniae*: Progressive Spread and Four-Year Period of Observation in a Cardiac Surgery Division

**DOI:** 10.1155/2015/871947

**Published:** 2015-05-04

**Authors:** Fortunata Lombardi, Paola Gaia, Rea Valaperta, Maria Cornetta, Milvana Rosa Tejada, Luca Di Girolamo, Alessandra Moroni, Federica Ramundo, Alessio Colombo, Massimiliano Valisi, Elena Costa

**Affiliations:** ^1^Research Laboratories-Molecular Biology, IRCCS Policlinico San Donato, San Donato Milanese, 20097 Milan, Italy; ^2^Clinical Microbiology Laboratory, IRCCS Policlinico San Donato, Milan, Italy; ^3^Service Lab Fleming Research, Milan, Italy

## Abstract

Frequent use of carbapenems has contributed to the increase to* K. pneumoniae* strains resistant to this class of antibiotics (CRKP), causing a problem in the clinical treatment of patients. This investigation reports the epidemiology, genetic diversity, and clinical implication of the resistance to drugs mediated by CRKP in our hospital. A total of 280* K. pneumoniae* strains were collected; in particular 98/280 (35%) were CRKP. Sequencing analysis of CRKP isolated strains showed that 9/98 of MBL-producing strains carried the *bla*_VIM-1_ gene and 89/98 of the isolates were positive for *bla*_KPC-2_. Antimicrobial susceptibility tests revealed a complete resistance to third-generation cephalosporins and a moderate resistance to tigecycline, gentamicin, and fluoroquinolones with percentages of resistance of 61%, 64%, and 98%, respectively. A resistance of 31% was shown towards trimethoprim-sulfamethoxazole. Colistin was the most active agent against CRKP with 99% of susceptibility. Clonality was evaluated by PFGE and MLST: MLST showed the same clonal type, ST258, while PFGE analysis indicated the presence of a major clone, namely, pulsotype A. This finding indicates that the prevalent resistant isolates were genetically related, suggesting that the spread of these genes could be due to clonal dissemination as well as to genetic exchange between different clones.

## 1. Introduction

In patients undergoing cardiac surgery, healthcare-associated infection often represents a dramatic event, with a consequent prolonged hospitalization and increased mortality [1]. The most common microorganisms causing infection are gram positive bacteria, with* Staphylococcus* spp. being the most frequent, followed by* Enterococcus* spp.,* Pseudomonas aeruginosa*,* Escherichia coli*, and* Acinetobacter baumannii* [2]. During the last decade, various multidrug-resistant organisms (MDRO) emerged, constituting a new challenge for pharmacological treatment and the implementation of infection control practices.

In the class of Enterobacteriaceae,* Klebsiella pneumoniae* was widespread in hospital environments and their diffusion was being facilitated by their being a normal colonizer of the gastrointestinal tract and by their having a high efficiency of resistant strains selection. This resistance was due to chromosomal mutations and to the presence of many transmissible plasmids. During outbreaks, a high number of carriers have been reported among patients and personnel in these wards, due to the colonization of hands and nasopharynx [3].

*Klebsiella pneumoniae* has become progressively resistant to penicillin, aminoglycosides, extended-spectrum *β*-lactamase, and fluoroquinolones. In the 2000s, when carbapenems represented the last resort for the treatment of infection caused by extended-spectrum *β*-lactamase (ESBL) producing bacteria, strains producing carbapenemases encoded by mobile elements arose, with a variety of enzymes produced.

The clinically most important* Klebsiella pneumoniae* carbapenemase (KPC) belongs to the class A enzymes encoded by *bla*_KPC_ genes and the class B metallo-*β*-lactamases (MBL), mainly NDM (New Delhi metallo-beta-lactamase) and VIM (Verona integron-encoded metallo-beta-lactamase). Both of these classes of A and B enzymes have been implicated in the rapid dissemination of the MDRO carbapenem-resistant* Klebsiella pneumoniae* (CRKP); this species seems to represent a “reservoir” of resistance, transmittable to other enterobacteria, including* Escherichia coli* and* Enterobacter* spp. [4, 5]. CRKP strains are implicated in nosocomial outbreaks and cause serious infections in intensive care units (ICUs); the respiratory tract was the most common site of infection [6], but also catheter related infection, surgical site, and urinary infection are reported [7].

Heart surgery patients are frequently affected by important comorbidity (diabetes mellitus, renal disease, respiratory disease, etc.) and are exposed to several healthcare-associated risk factors, such as mechanical ventilation, parenteral nutrition, arterial and central lines, and urinary catheter [8]. Some of these patients have additional risk factors for postoperative infection, like hemodialysis, blood transfusion, and readmission to the ICU [9].

Diagnosis and hospital management of CRKP infection represent a major problem in hospitals worldwide. For these patients, time is a critical parameter for the successful implementation of treatment and this should be considered in relation to the time required by the microbiology laboratory to confirm the bacteriological identification and the antimicrobial pattern, to support the decision on antibiotic therapy. This information is important for designing and implementing interventions aiming at reducing the spread of antimicrobial resistance [10].

The emergence of* K. pneumoniae* resistance to carbapenems is well documented in several studies [11–17] but more information about the molecular characterization of CRKP and their antimicrobial resistance patterns and molecular typing could be keys for the epidemiological investigation of hospital-onset CRKP infection. The Policlinico San Donato is an IRCCS (Scientific Institute for Research, Hospitalization and Health Care), with a particular attention on cardiovascular diseases. In the cardiovascular centre “E. Malan,” five operating rooms and 102 beds for patients of Heart Surgery (41 beds), Pediatric Heart Surgery (33 beds), and Postoperative Intensive Care (28 beds) Divisions are functional. Between 2010 and 2013, 1450 heart surgical interventions have been performed, representing more than 20 percent of all cardiosurgery activities in the Italian region of Lombardy. Therefore, the principal objective of the current work was to study the molecular epidemiology of* K. pneumoniae* strains circulating in this institution between 2010 and 2013. Secondly, all* K. pneumoniae* strains were characterized phenotypically by antimicrobial susceptibility detection and genotypically by multilocus sequence typing (MLST) and by pulsed-field gel electrophoresis (PFGE) analysis. The respective allelic variants were determined to understand the clonal relationship and to better control their dissemination.

## 2. Materials and Methods

### 2.1. Laboratory Data

#### 2.1.1. Microbiologic Analysis

Between February 2010 and December 2013, a total of 280 nonduplicate* K. pneumoniae* strains were isolated from patients hospitalized in the Cardiac Surgery ward of the IRCCS Hospital of San Donato, including Postoperative Intensive Care, Cardiac Surgery for Adults, and Pediatric Cardiac Surgery. All strains were identified by Clinical Microbiology Laboratory and extracted from several biological sources such as blood, bronchoalveolar lavage (BAL), uroculture, wound swab, and other sources. Species identification and susceptibility testing were performed by the semiautomated systems VITEK 2 (bioMerieux, France). Minimum inhibitory concentrations (MIC) of meropenem and imipenem were confirmed by Etest (AB Biodisk, Sweden) on Mueller-Hinton agar. Susceptibility results were interpreted in accordance with clinical guidelines of the European Committee on Antimicrobial Susceptibility Testing (EUCAST). All strains with a meropenem level greater than 0.5 *μ*g/mL were subject to genotypic determination; carbapenemases detection was confirmed by a modified Hodge test [11] and by a Synergy test that combined disk for meropenem (10 *μ*g), tested with meropenem + dipicolinic acid (100 mg/mL in DMSO) (MRPDP) and meropenem + 3-aminophenylboronic acid (60 mg/mL in DMSO) (MR+BO) (Rosco Diagnostica, Denmark).

#### 2.1.2. Molecular Analysis

For chromosomal DNA extraction, several colonies were suspended in about 50 *μ*L of sterile distilled water and heated to 95°C for ten minutes. Each sample was then centrifuged for five minutes at 2500 ×g. After centrifugation, DNA concentration was assessed by spectrophotometry and stored at −20°C.

Primer specific for carbapenemase genes (*bla*_KPC_, *bla*_VIM_, and *bla*_IMP_) and polymerase chain reaction (PCR) amplification conditions were performed as previously described [18].

Sequencing reactions were performed by the Big Dye Terminator v3.1/1.1 cycle sequencing kit (Applied Biosystems, Foster City, CA, USA) and by an automated ABI PRISM 3100 genetic analyzer sequencer (Applied Biosystems). The nucleotide sequences were analyzed using software by the National Center for Biotechnology Information (http://www.ncbi.nlm.nih.gov/).

We investigated clonal relatedness for epidemiological comparison using MLST and PFGE.

The genotype of carbapenem-resistant strains was determined by MLST analysis, performed as described by Diancourt et al. [19]. Fragments of seven housekeeping genes* rpoB*,* gapA*,* mdh*,* pgi*,* phoE*,* infB*, and* tonB* were obtained from chromosomal DNA and directly sequenced. Allelic profiles and sequence types (STs) were designated at the website http://www.pasteur.fr/recherche/genopole/PF8/mlst/Kpneumoniae.html.

We performed a second genotype analysis using PFGE of* Xba*I-digested total DNA with gene path system (Bio-Rad, Hercules, CA, USA). Band profiles were inspected by Fingerprinting II software (Bio-Rad). The clonal relationship was interpreted according to Tenover criteria [20].

#### 2.1.3. Statistical Analysis

We used the Student's *t*-test for statistical analysis. *P* values of <0.05 were considered to indicate statistical significance.

## 3. Results

### 3.1. Department and Material Distribution of* K. pneumoniae* Strains

**Figure 1:**
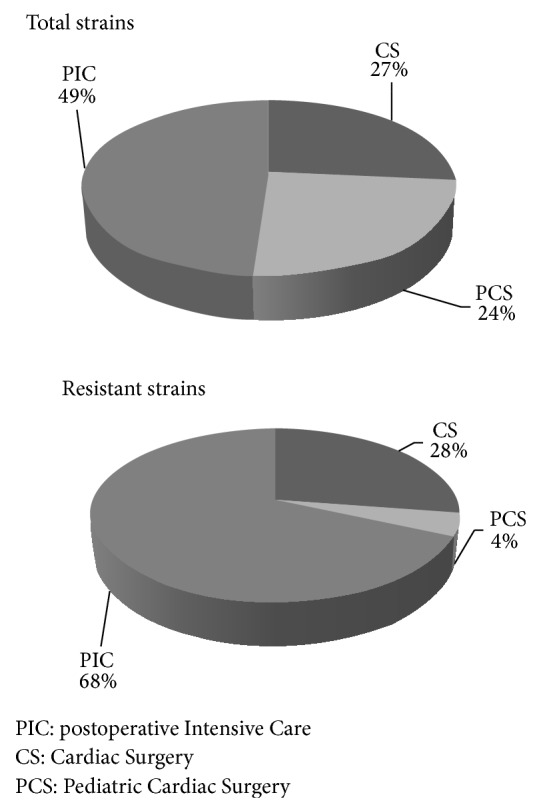
Distribution of* K. pneumoniae* isolates by Cardiac Surgery department. The above pie chart shows the total distribution of* K. pneumoniae* isolates by department. The pie chart below shows the distribution of* K. pneumoniae* resistant isolates by department.

Between February 2010 and December 2013, a total of 280 consecutive nonreplicate clinical isolates of* K. pneumoniae* were isolated from patients hospitalized in the Cardiac Surgery ward of the IRCCS Hospital of San Donato Milanese, including Postoperative Intensive Care (*n* = 137; 49%), Cardiac Surgery for Adults (*n* = 75; 27%), and Pediatric Cardiac Surgery (*n* = 68; 24%) (Figure 1, above).

**Figure 2:**
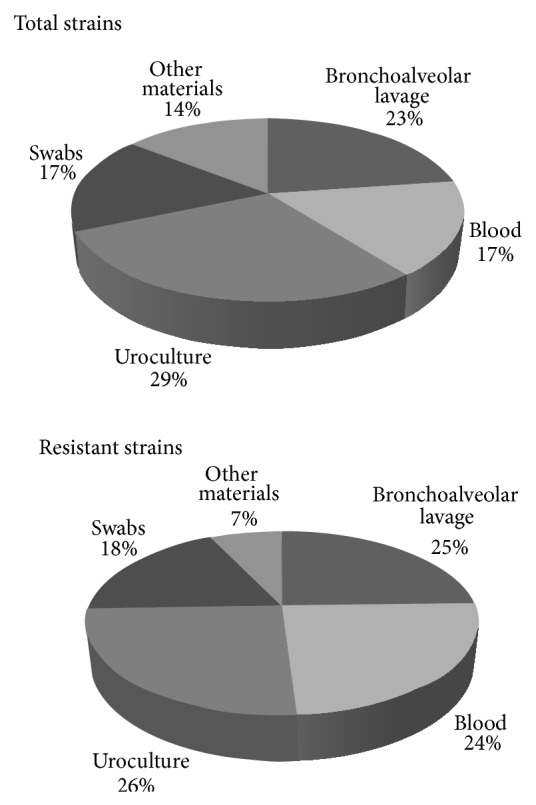
Distribution of* K. pneumoniae* isolates by type of biological materials. The above pie chart shows the total distribution of* K. pneumoniae* isolates by several biological materials. The pie chart below shows the distribution of* K. pneumoniae* resistant isolates by several biological materials.

*K. pneumoniae* isolates were extracted from several clinical samples: uroculture 29% (*n* = 82), BAL 23% (*n* = 64), blood 17% (*n* = 46), swabs 17% (*n* = 48), and other sources 14% (*n* = 40) (Figure 2, above). Uroculture and bronchoalveolar lavage were the most commonly infected by* K. pneumoniae* strains. Of the total* K. pneumoniae* strains, 98/280 (35%) isolates were resistant to carbapenems (CRKP) and, of the remaining 182 isolates, 36/280 (13%) were susceptible to all drugs while 146/280 (52%) were ESBL (resistant to third-generation cephalosporins).

67 of 137 strains derived from Postoperative Intensive Care (49%) were resistant to carbapenems. For what concerns Cardiac Surgery for Adults, a similar incidence of resistance was observed (27/75; 41%) whereas only 4 out of 68 strains derived from Pediatric Cardiac Surgery (5%) were found to be CRKP.

In Figure 1 (below) is reported the distribution of CRKP by ward: Postoperative Intensive Care (*n* = 67; 68%), Cardiac Surgery for Adults (*n* = 27; 28%), and Pediatric Cardiac Surgery (*n* = 4; 4%). In Figure 2 (below) is reported the distribution by biological source: urine, bronchoalveolar lavage, and blood were the most frequent sources of CRKP strains with an incidence of 26% (*n* = 25), 25% (*n* = 24), and 24% (*n* = 24), respectively. These resistant isolates that exhibited reduced carbapenems susceptibility were selected as the object of this study.

### 3.2. Molecular Characterization of CRKP Strains

To gather information on the molecular epidemiology of the CRKP strains diffused in Northern Italy, the 98 isolates were characterized by MLST and PFGE genotyping and their allelic variants were determined.

PCR assays performed for the 98 isolates collected during the four years exhibited reduced susceptibility to carbapenems. The resulting amplifications demonstrated that 9/98 MBL-producing isolates carried a *bla*_VIM_ gene and 89/98 isolates were positive with the primer specific for *bla*_KPC_. None of the total isolates contained *bla*_IMP_ or produced either VIM or KPC carbapenemase. Sequencing analysis allowed identifying VIM-positive isolates as type 1 and KPC-positive as type 2.

Genetic relationship between all the resistant isolates was investigated using MLST. The most prevalent profile of CRKP was ST258 with the allelic profile of 3-3-1-1-1-1-79.

In addition, PFGE detected 3 pulsotypes when a similarity cut-off value of 80* *% was implemented. PFGE profiles indicated the presence of a major pulsotype, namely, pulsotype A, followed by pulsotype B and pulsotype C.

The A and C clones include VIM-producing and KPC strains, while the clone B includes only VIM-producing carbapenem-resistant* Klebsiella pneumoniae*. The clones A and C harbored both resistance determinants (*bla*_VIM_ and *bla*_KPC_), while the B clone harbored only the gene *bla*_VIM_.

The pulsotype A was found for the first time in January 2010 in the Postoperative Intensive Care and it was observed that it still persists in this care unit and in Cardiac Surgery for Adults. The pulsotype B was identified in the month of February 2010 in only Postoperative Intensive Care, while the spread of the clone C derived from Pediatric Cardiac Surgery and Postoperative Intensive Care.

### 3.3. Temporal and Age Group Distribution of CRKP Strains

**Figure 3:**
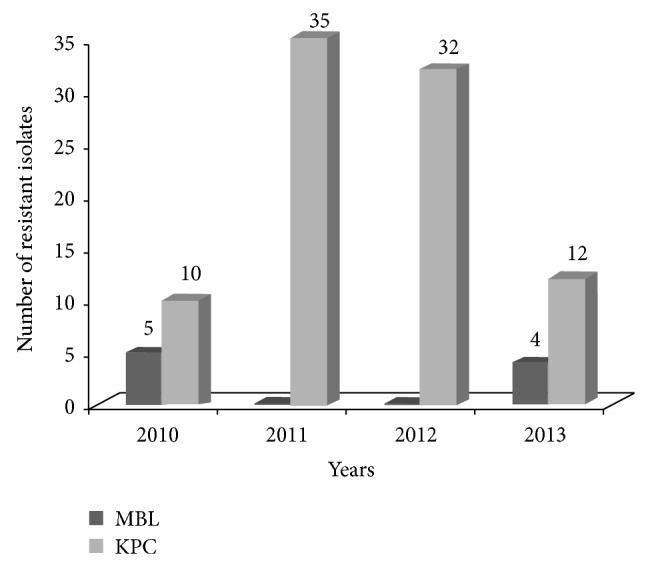
Temporal distribution of carbapenem-resistant* K. pneumoniae* isolates during the study period (February 2010–December 2013).

The epidemic curve revealed three phases: period 1 (February 2010 to December 2010) during which the first cases of resistant strains began to emerge: 5 isolates were MBL-KP and 10 strains were KPC-KP; period 2 (January 2011 to December 2012) during which we observed a dramatic increase in KPC-KP strains which persisted until the end of 2012. In particular, we identified 35 KPC-KP strains in 2011 and 32 KPC-KP strains in 2012. Period 3, during which the spreading subsided, presented 4 cases of MBL-KP and 12 isolates of KPC-KP strains (Figure 3).

The age of the patients contributing the CRKP isolates varied between 2 months and 86 years and the gender ratio male : female was 2 : 1.  Table 1 shows the distribution of resistant isolates based on the age of the patients. Most of the MBL-KP strains (6/9) were found in patients under 5 years of age while 56/89 were KPC-KP isolates from patients with an age ranging between 66 and 80 years. The male group constituted the majority of the patients except in the case children.

### 3.4. Antimicrobial Susceptibility Profile in CRKP Strains

Susceptibility testing of the 182 carbapenems susceptible strains revealed that 20% (*n* = 36) were susceptible to all drugs tested, while 80% (*n* = 146) were nonsusceptible to third-generation cephalosporin (data not shown). In February 2010, we had the first case of MBL-KP isolate resistant to meropenem (MIC > 8 mg/L), isolated from the respiratory tract of a child who was admitted to the Pediatric Cardiac Surgery ward. After one month, KPC-KP spread rapidly becoming increasingly prevalent, while only a few cases of MBL-KP isolates were recorded between 2010 and 2013. A wide spectrum of resistance patterns to most classes of antibiotics was shown in the CRKP strains (Table 2).

Resistance to imipenem, meropenem, and ertapenem in all 98 isolates was variable (MICs ranging from 1 to >8 mg/L). Antimicrobial susceptibility tests revealed a complete resistance (100%) to third-generation cephalosporins (cefotaxime, cefepime, and ceftazidime MICs > 4 mg/L). A moderate resistance, between 60% and 65%, was shown to tigecycline (MICs > 2 mg/L) and gentamicin (MIC > 4 mg/L), while a higher resistance, of 96%, was shown to fluoroquinolones: ciprofloxacin and levofloxacin (MICs > 1 mg/L and MICs > 2 mg/L, resp.). Resistance of 31% was present toward trimethoprim-sulfamethoxazole. Colistin was the most effective agent against CRKP with 99% of susceptibility (MICs < 2 mg/L).

In that order, among CRKP isolates, tigecycline resistance increased from 60% (9/15) in 2010 to 91% (29/32) in 2012 and this percentage has remained unchanged throughout 2013.

On the other hand, we observed a decrease in CRKP strains resistant to gentamicin and trimethoprim-sulfamethoxazole going from a rate of about 69% (22/32) in 2012 to 43% (7/16) in 2013 and from a rate of 41% (13/32) in 2012 to 25% (4/16) in 2013, respectively. Notably, only one KPC-KP strain was resistant to colistin and it appeared in 2013.

The susceptibility to fluoroquinolones was observed only in MBL-KP strains and it was of 4%. Most of the MBL-KP isolates were found in the bronchoalveolar lavage in the Pediatric Cardiac Surgery ward (4/9) while KPC-KP strains were more prevalent in urocultures (26/89) of which 62% (16/26) were isolated in the Postoperative Intensive Surgery ward.

In addition, the predominant resistant antibiotyping was the same: in particular, 44% (4/9) of the MBL-KP and 21% (19/89) KPC-KP strains were characterized by a resistance to gentamicin, trimethoprim-sulfamethoxazole, tigecycline, fluoroquinolones, and third-generation cephalosporins and were susceptible to colistin.

## 4. Discussion

KPC spread rapidly in our hospital, becoming increasingly prevalent between 2011 and 2012, while in this period VIM-producing isolates disappeared. Since mid-2013, the incidence of KPC isolates has been gradually declining, whereas VIM producers reappeared in a few cases, at a much lower rate. We PCR-amplified and sequenced the DNA of carbapenemase genes and used MLST or PFGE to examine the molecular epidemiology to better characterize the dissemination of these isolates. The MLST and PFGE data aided in identifying the possible route of dissemination of the isolates; MLST showed that all CRKP strains belonged to the same clonal type, ST258, while PFGE revealed that there were three different strains and most of the isolates were owing to the same clone A. The other sample was genetically unrelated among the common clone. Though all the strains were phenotypically the same, PFGE revealed genetical discrepancy between the strains.

Microbiological investigation showed multidrug-resistant profiles between MBL-KP and KPC-KP strains, even if the prevalent resistant antibiotyping was the same. Fortunately, a high proportion of CRKP isolates still showed* in vitro* susceptibility to aminoglycosides (37%), tigecycline (39%), and trimethoprim-sulfamethoxazole (69%). In addition, they presented complete coresistance to the fluoroquinolones and to third-generation cephalosporins. A colistin-resistant CRKP isolate was also identified from a patient with a history of colistin therapy. This strain was resistant to all drugs tested. A double carbapenem regimen was employed to treat this patient.

The infections caused by multidrug resistant* K. pneumoniae* have been reported with an increasing frequency in the Intensive Care Unit and in the Cardiac Surgery Unit. CRKP strains constitute an emergent public health hazard and they are associated with a significant morbidity and mortality [21]. Many studies have focused on the risk factors for acquiring this pathogen and the impact of bacteraemia on the outcome. Our results are concordant with the previous studies: KPC type 2 is present in the majority of clinical specimens and accounts for most of the epidemic outbreaks. KPC-2 appears to be more predominant worldwide, with outbreaks arising in Europe and especially in Italy [22]. Starting from 2010, KPC-2 was detected in different regions of Italy. Interestingly, we found that the predominant type of* K. pneumoniae* associated with KPC and MBL production was ST258. It is increasingly recognized that ST258 is the predominant clone of resistant isolates all over the world and has caused outbreaks in many countries [23, 24].

The fact that CRKP isolates belonged to ST258 and were acquired in our hospital indicates the possibility that the spread of these genes could be due to clonal dissemination as well as to genetic exchange between different clones. However, the failure to find a common environmental reservoir indicated that patient-to-patient transmission may be the main mechanism of CRKP spread in our hospital. Frequent bed transfers of patients, particularly after isolation of CRKP, combined with the lack of adequate preventive measures, might have facilitated this process.

As of today, few safe and practical therapeutic options remain for patients infected with KPC producers. Many clinicians have resorted to the use of tigecycline, polymyxins, and the few remaining aminoglycosides [25]. Tigecycline consistently shows* in vitro* activity against most isolates of KPC-producing organisms. In any case, some shortcomings limit its use in monotherapy regimens: due to its high volume of distribution, for example, tigecycline does not always achieve high serum or urinary concentrations after infusion at standard doses [26]. Furthermore emergent resistance to tigecycline has been reported; thus, clinicians should remain vigilant for clinically refractory infections when tigecycline is being used. Despite these limitations, tigecycline has been successfully used to treat and cure patients infected with KPC-producers. Another class of antibiotics that has been successfully used to treat CRKP strains is polymyxins, such as colistimethate and colistin. These drugs cause very little toxicity (namely, mild dose-dependent renal toxicity) and have shown good efficacy in several studies. According to these, a significant finding of this report is that we observed only one strain resistant to colistin in all CRKP isolates. This may be due to the restricted use of the drug in our hospital. Indeed it is employed almost exclusively to treat MDR infections caused by CRKP or* Acinetobacter Baumannii*. It is suggested to utilize polymyxins in combination with other antibiotics [27–30].

Combination therapy improves the chances of cure in highly resistant infections, due to a synergistic effect and to the minimization of prolonged therapy which could abet resistance spreading [31]. It is noteworthy that in some cases colistin represents the “last-line” therapeutic drug against CRKP pathogens. Flowcharts for selecting mainstream and adjuvant therapy against gram-negative bacteria are described by Zavascki et al. [32]; these guidelines are recently adopted also in our departments.

With regards to other antibiotics tested in the study (Table 2), we found high resistance rates for cephalosporins, chinolons, and gentamicin. This was not surprising taking into account the selective pressure due to the frequent use of these drugs.

On the contrary, a discrete trimethoprim-sulfamethoxazole (SXT) sensibility characterized our strains. But, due to its high restitution volumes and sodium content, SXT represents an inconvenient drug for intravenous administration. For this reason, SXT is infrequently used in surgery and intensive care units like those considered in this work. Further studies are advocated in order to evaluate the real consistency of this finding.

Finally, we think that the significant reduction in CRKP isolates observed from 2011 onwards is important. This coincided with a strong enhancement, in our institution, of the recommendations for prevention of healthcare-associated infections that include performance of contact precautions in addition to standard precautions (hand hygiene, use of gloves, and protective coating), isolation of the infected patients in separate rooms with designated nursing staff; promotion of more targeted antibiotic therapy, minimization of the invasive medical devices use, and rectal swab screening for all new admitted patients with risk factors for CRKP in the cardiosurgery area.

These results and the decline in the discovery of new effective drugs suggest that the prudent and conservative use of available active agents combined with good control practices still represent the keys to curtailing the spread of this dramatic antimicrobial resistance.

## Figures and Tables

**Table 1:** Age groups and sex-wise details of patients from whom carbapenem-resistant *K. pneumoniae* was isolated.

Age years	Resistant strains		MBL		KPC	
	M	F	M	F	M	F
<5 y	3	5	2	4	1	1
6–25 y	2	1	1	0	1	1
26–45 y	2	0	0	0	2	0
46–65 y	9	5	0	0	9	5
66–80 y	38	19	1	0	37	19
>80 y	6	8	0	1	6	7

**Table 2:** Antimicrobial resistance profile of clinical isolates of carbapenem-resistant *K. pneumoniae *from our hospital (February 2010–December 2013).

Antibiotic/MIC (*μ*g/mL)	Total	Carbapenemase	
	no (%)	MBL/no	KPC/no
IMP			
>4	98 (100)	9	89
MER			
>8	98 (100)	9	89
EPT			
>8	98 (100)	9	89
GEN			
>4	63 (64)	6	57
SXT			
>40	30 (31)	4	26
CS			
>2	1 (1)	—	1
TGC			
>2	60 (61)	6	54
FEP			
>4	98 (100)	9	89
CTX			
>4	98 (100)	9	89
CAZ			
>4	98 (100)	9	89
CIP			
>1	96 (98)	7	89
LVX			
>2	96 (98)	7	89

IMP: imipenem; MER: meropenem; EPT: ertapenem; GEN: gentamicin; SXT: trimethoprim-sulfamethoxazole; CS: colistin; TGC: tigecycline; FEP: cefepime; CTX: cefotaxime; CAZ: ceftazidime; CIP: ciprofloxacin; LVX: levofloxacin.
